# mHealth intervention for multiple lifestyle behaviour change among high school students in Sweden (LIFE4YOUth): protocol for a randomised controlled trial

**DOI:** 10.1186/s12889-021-11446-9

**Published:** 2021-07-16

**Authors:** Marcus Bendtsen, Anna Seiterö, Preben Bendtsen, Hanna Henriksson, Pontus Henriksson, Kristin Thomas, Marie Löf, Ulrika Müssener

**Affiliations:** 1 Department of Health, Medicine and Caring Sciences, Linköping University, 581 83 Linköping, Sweden; 2 Department of Medical Specialist, Motala, Sweden; 3 Department of Biosciences and Nutrition, Karolinska Institutet, Stockholm, Sweden

**Keywords:** Telemedicine, Multiple behaviour, mHealth, High school students, Randomised controlled trial

## Abstract

**Background:**

National surveys in Sweden demonstrate that the majority of young people do not engage in health promoting behaviours at levels recommended by the Public Health Agency of Sweden. The objective of this study is to estimate the effectiveness of a novel mHealth intervention named LIFE4YOUth, which targets multiple lifestyle behaviours (alcohol, diet, physical activity, and smoking) among high school students in Sweden.

**Methods:**

A 2-arm parallel groups single blind randomised controlled trial (1:1) will be employed to estimate the effectiveness of the novel mHealth intervention. Students will be recruited at high schools throughout Sweden, and will be included if they fulfil one of six criteria relating to unhealthy behaviours with respect to alcohol, diet, physical activity and smoking. Eligible participants will be randomised to either receive the novel intervention immediately, or to be placed on a waiting list for 4 months. The intervention consists of a combination of recurring screening, text messages, and an interactive platform which is adaptable to individual preferences. Outcome measures with respect to alcohol, diet, physical activity and smoking will be assessed through questionnaires at 2 and 4 months post randomisation.

**Discussion:**

The findings of this trial could be generalised to a diverse high-school student population as our recruitment encompass a large proportion of schools throughout Sweden with various educational profiles. Furthermore, if effective, the mHealth intervention has good potential to be able to be scaled up and disseminated at high schools nationally.

**Trial registration:**

Registered prospectively on 2020-05-20 in ISRCTN (ISRCTN34468623).

**Supplementary Information:**

The online version contains supplementary material available at 10.1186/s12889-021-11446-9.

## Background

The “big four” health risk behaviours of excessive alcohol consumption, poor diet, physical inactivity and smoking, significantly contribute to the global burden of disease. The association between health risk behaviours and non-communicable diseases such as cardiovascular disease, cancer, respiratory disease, and type II diabetes, has been consistently shown in research [1–3].

As adolescence is a critical period of life when health-related behaviours are set for adulthood, it is crucial that adolescents adopt healthy behaviours. National surveys in Sweden demonstrate that the majority of young people do not engage in health promoting behaviours at levels recommended by the Public Health Agency of Sweden. For instance, the majority of young people fail to meet national recommendations on the intake of fruit and vegetables and level of physical activity [4–6]. Furthermore, while alcohol consumption has declined among young people, heavy episodic drinking and associated risks continues to be a problem [7]. Finally, smoking is still highly prevalent among young adults exhibiting a major health risk [7, 8].

Over the past decade, research has shown that mobile phone-based interventions (mHealth interventions) may be effective in promoting healthy lifestyles and supporting individuals in behaviour change [9–18]. To date, most of these mHealth interventions, such as text messaging or smart phone applications, only target one or two health risk behaviours, for instance nutrition and/or physical activity or smoking cessation [19]. However, health risk behaviours typically cluster and most individuals report at least two health risk behaviours at the same time [20]. Furthermore, engaging in multiple risk behaviours has been found to correspond with an increased risk greater than the sum total risk of individual behaviours [21–23]. Thus, the development of effective interventions that target multiple changes in behaviour could potentially have a valuable impact on individuals’ health and may be the way forward for the mHealth research field.

Despite the perceived benefits, there are few studies of interventions targeting “the big four”, especially in adolescent and young adult populations. A meta-analysis [24] examined the effects of text message-based interventions targeting tobacco use and alcohol consumption within a young adult population. Out of 14 studies, five reported a positive effect on substance-use. Another systematic review investigated the effects of mHealth on preventative behaviours related to “the big four”, but also included other health behaviours such as oral health and contraceptive use. The authors concluded that although eight out of 19 studies showed significant improvements in preventative behaviours, most studies were low to moderate in quality [25]. Thus, we lack evidence on the effects of mHealth interventions targeting multiple behaviours related to alcohol, diet, physical activity and smoking among adolescents and young adults.

### Objectives

The objective of this study is to estimate the effectiveness of a novel mHealth intervention named LIFE4YOUth, which targets multiple lifestyle behaviours (alcohol, diet, physical activity, and smoking) among high school students in Sweden.

LIFE4YOUth is part of the MoBILE research program (funded by Forte 2018–01410; PI: ML) aiming to promote non-risky drinking, healthy eating, physical activity and smoking cessation [26]. The research program will develop, evaluate, and implement mHealth interventions among different target populations throughout the lifespan, including pregnant women, pre-school children, young adults, as well as clinical and healthy adult populations.

A 2-arm parallel group (1:1) single blind randomised controlled trial will be employed, where participants will be randomised to an intervention or a control group. The intervention group will be given immediate access to the novel intervention, while the control group will be given general health information and be placed on a waiting list. The key objectives of the trial are to:

1. Estimate the effectiveness of the intervention on individual lifestyle behaviours with respect to:
  - Weekly alcohol consumption and number of episodes per month of heavy drinking.
  - Weekly consumption of sugary drinks and average daily fruit and vegetable consumption.
  - Weekly moderate to vigorous physical activity.
  - Smoking.
2. Estimate to which degree the total effects are mediated through psychosocial factors.
3. Detect interactions among lifestyle behaviour change, e.g. those who stop smoking may also reduce their alcohol consumption, and the degree to which this is moderated by access to the intervention.
4. Investigate acceptability of the novel intervention in terms of users’ experience.
5. Investigate reactions and actions among participants allocated to the control condition.

## Methods

**Fig. 1:**
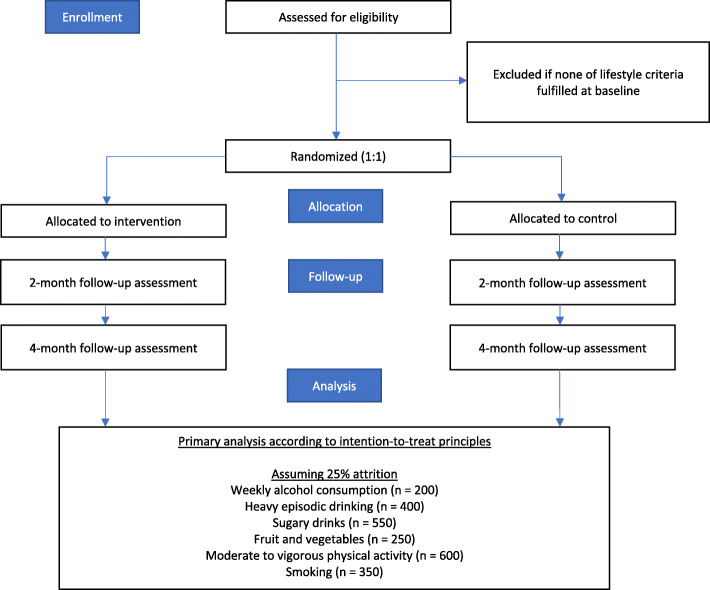
Trial design depicted in a CONSORT flow diagram

**Fig. 2:**
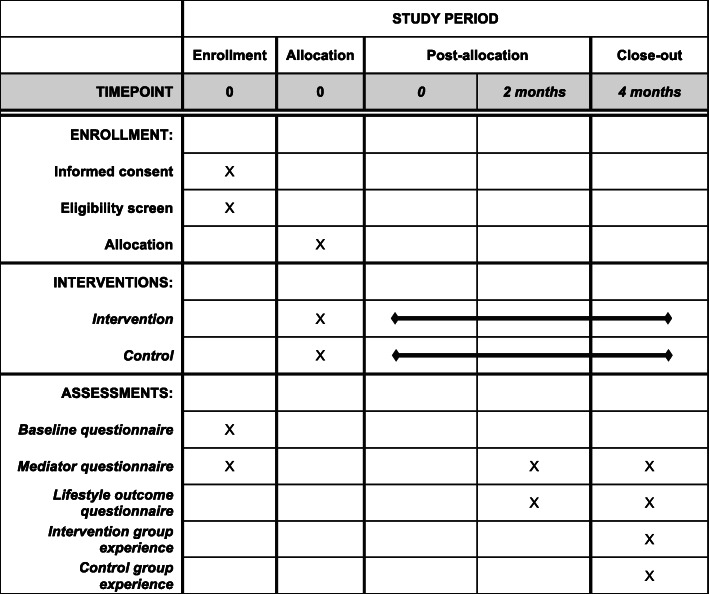
SPIRIT figure depicting participant timeline

A 2-arm parallel groups single blind randomised controlled trial (1:1) will be employed to estimate the effectiveness of the novel intervention. A flow diagram of the trial design can be found in Fig. 1, and a trial participant timeline is presented in Fig. 2. This protocol follows the SPIRIT guidelines [27].

### Study setting, recruitment and eligibility

Participating high schools (approximately 300 high schools with approximately 80,000 students in total) will recruit students to the trial using: printed advertising (posters and leaflets), digital advertising (email, school website, app), and school staff (teachers, mentors, and/or school health centers). Participants will initially be recruited over a 6-month period, with additional 3-month periods added until the required number of participants has been reached (see Power Calculations). Recruitment will not extend past 24 months, regardless of number of participants recruited, and will commence in September 2020.

Students will register their interest by sending a text message to a dedicated telephone number (included in all information materials). In response, students will receive a text message with a hyperlink to a web page presenting trial information and will be asked to give informed consent to participate. All students who consent will immediately be asked to complete an online baseline questionnaire (see Additional file 1), which will also be used to assess eligibility for the trial.

Students will be included in the trial if they fulfil at least one of six conditions, which are related to the primary outcomes of the trial. The conditions are:

- **Weekly alcohol consumption:** Consumed 10 or more standard drinks of alcohol the past week. A standard drink of alcohol is in Sweden defined as 12 g of pure alcohol.
- **Heavy episodic drinking:** Consumed 4 or more standard drinks of alcohol on a single occasion at least once in the past month.
- **Fruit and vegetables:** Consumed less than 500 g of fruit and vegetables on average per day the past week.
- **Sugary drinks:** Consumed 3 or more units of sugary drinks the past week. One sugary drink unit is defined as approximately 33 cl.
- **Moderate to vigorous physical activity:** Spent less than 420 min on moderate to vigorous physical activity in the past week (ie. approximately 60 min per day).
- **Smoking:** Having smoked at least one cigarette the past week.

Students will be explicitly excluded if they do not fulfil any of the criteria. There will be no age restriction, however the majority of students attending high school in Sweden are between 16 and 19 years of age. Also, the trial information and intervention will be entirely in Swedish and delivered to participants’ mobile phones, thus students who do not comprehend Swedish well enough to be able to sign up or who do not have access to a mobile phone will be implicitly excluded.

Students completing the baseline questionnaire (see Additional file 1) will automatically be checked for eligibility given their responses, and eligible participants will be randomly allocated to either the control or intervention condition. Participants in both the intervention and control group will be recommended to visit a national website (https://www.1177.se/liv--halsa/) for general lifestyle and health information.

### Control and intervention conditions

#### Intervention

The formative research process of developing the novel multiple mHealth intervention has been described in detail previously [28]. User requirements and usability of the intervention were investigated in terms of function, content, and design by using heuristic evaluations and usability tests. The participatory design resulted in in-dept knowledge regarding aspects of intervention content and structures that end-users considered important and guided further development of the final version of LIFE4YOUth [29]. The intervention aims to promote reduction in alcohol consumption, a healthy diet, increased physical activity, and smoking cessation during a 16-week period among high school students. The structure and content are based on current best practice gathered from scientific literature on health promotion interventions and health behaviour change. Fundamental theoretical constructs are behaviour change theories and social-cognitive models [30, 31], and research emphasizing the importance of the quality of the actual encounter in meetings [32].

**Fig. 3:**
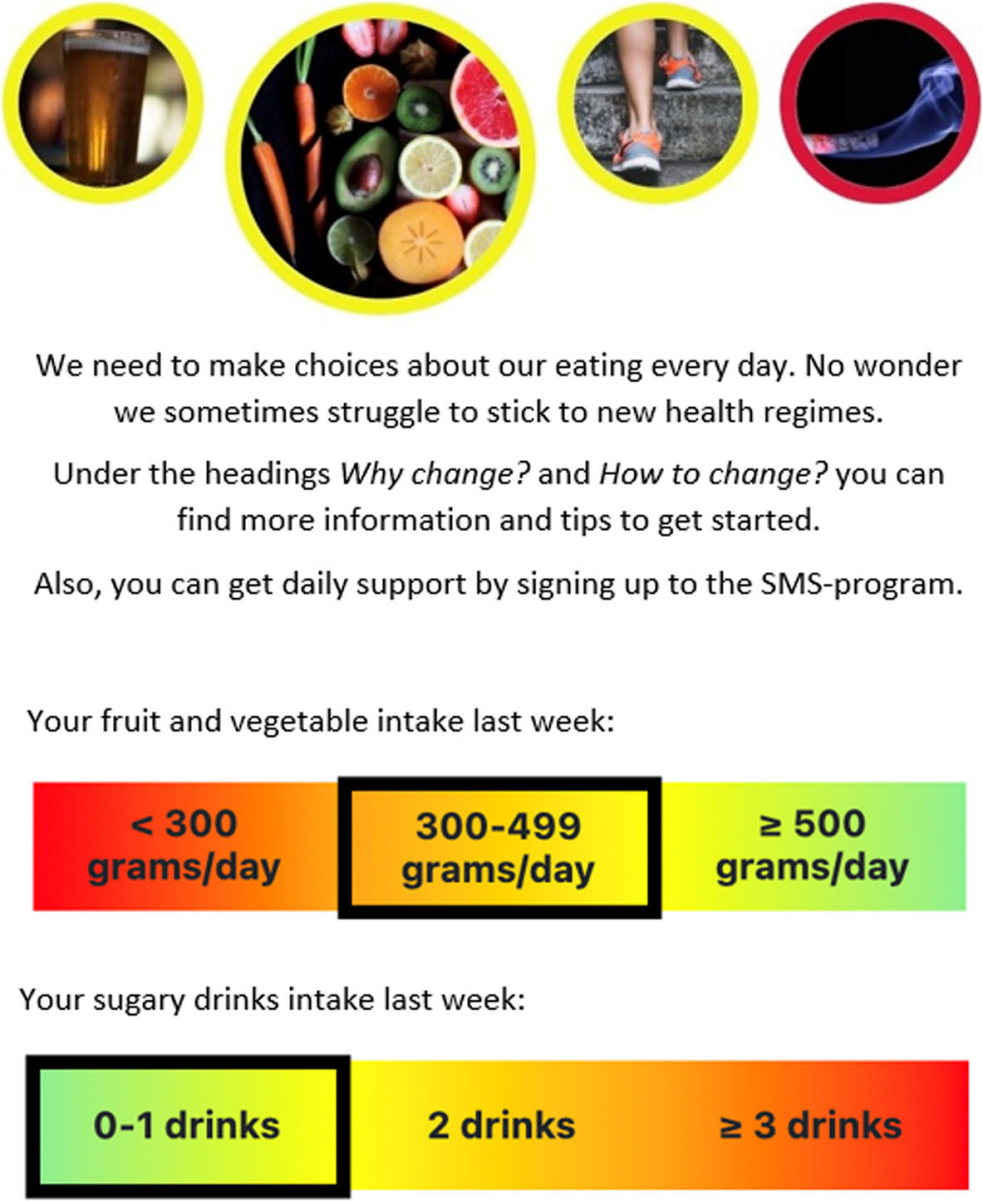
Screenshot from intervention showing feedback on past weeks fruit and vegetables and sugary drinks

Each week, users will receive a text message prompting a brief weekly screening that includes all four health behaviours, followed by feedback on individual screening results in comparison with national guidelines. The feedback will be delivered as a graduated coloured scale addressing each health behaviour where green indicates high agreement between reported health behaviours and national guidelines, yellow indicates that behaviour change can improve health and red indicates an increased risk for future health problems (see Fig. 3 for a screenshot). Users younger than 18 years will not receive coloured feedback in the module for alcohol but instead only a message that inform about recommendations to abstain from alcohol. All users will then access a personal interactive dashboard with pictures representing each health behaviour, with the ability to navigate as they wish between the four health behaviour modules. Users can hence choose to work with single or multiple health behaviours at a time.

Each behaviour module consists of two components: (1) content on *Why* to change behaviour and (2) *How* to change behaviour. The first component (*Why*) consists of:

- **Factual information**: Highlighting positive and negative consequences of the risk behaviour, outlined from an adolescent perspective. Factual information is given in text and video format as well as through images. Overall, an encouraging tone is used that acknowledges users’ autonomy and intends to promote self-reflexiveness regarding health behaviour change. Content is kept short, concise and easy to read, in accordance with preferences among adolescents [29].
- **Exercises:** Aiming to prompt users to explore their own reasons for behaviour change. Users are asked to identify reasons that represent their health behaviour change journey through a series of predefined statements (about 10 statements in total). For instance, “I want to be able to concentrate at school”, “I want to sleep better” or “I want to decrease the risk of disease”. Users can also enter their own reasons for behaviour change. All input will be automatically stored so that users can come back and review previous inputs. In addition, each health behaviour module (alcohol, diet, physical activity, and smoking) will include a calculator tool exercise. The calculator prompts users to for instance estimate how much money they spend on cigarettes or junk food. Finally, there is a module in which users are asked to pick between True or False given statements designed to challenge usual assumptions and provide insights and deeper understandings of the complexity of health behaviours.

**Fig. 4:**
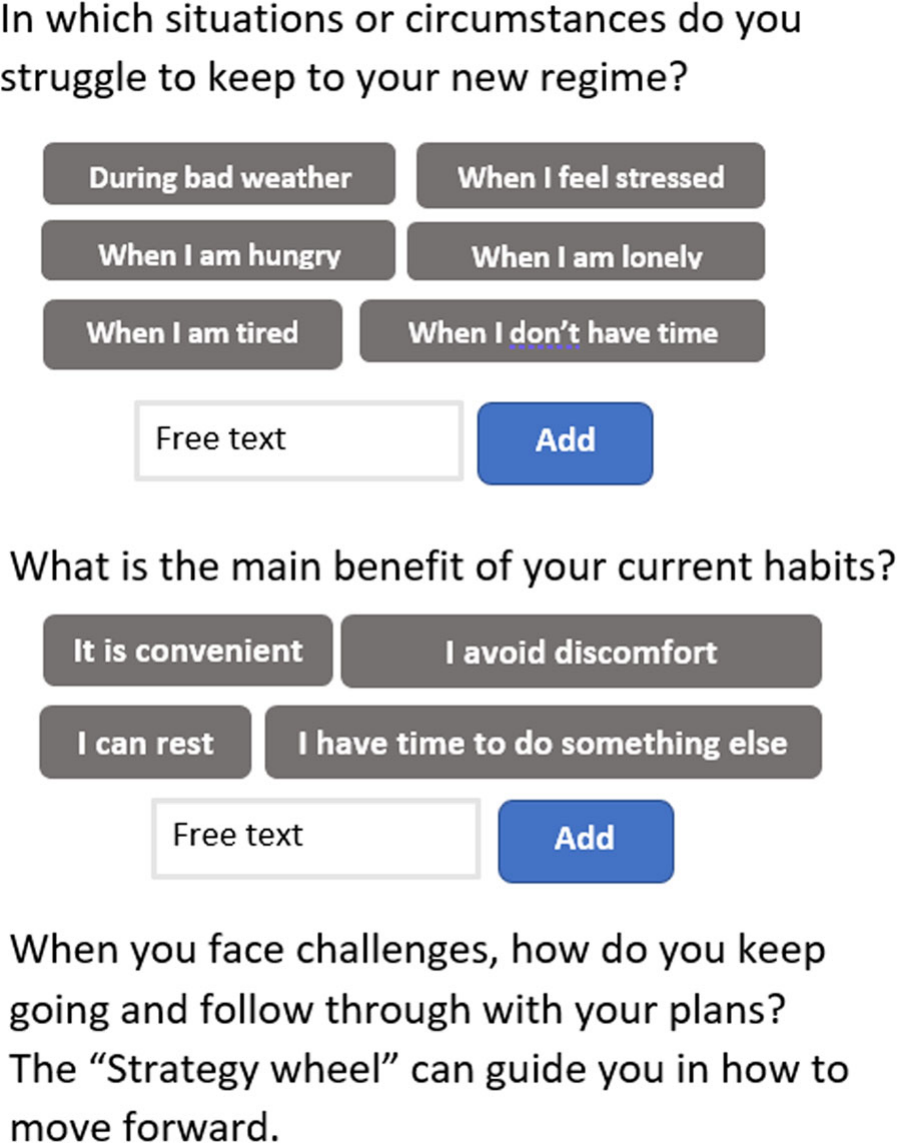
Screenshot from intervention showing module for creating a plan

The second component (*How*) consists of:

- **Factual information:** Which aims to boost users’ repertoire of strategies to replace and/or manage risk behaviours. For instance, “smoke less on each cigarette” or “explore your impressions without alcohol”.
- **Exercises:** Which include goal setting and making an action plan. To promote goal-setting skills and to support well-defined goals, users are provided with an example of what characterizes an effective goal (specific, measurable, achievable, realistic and timely goals). The action plan aims to stimulate self-reflection regarding behaviour triggers, unwanted habits, and perceived advantages about the risk behaviour (see Fig. 4 for a screenshot). A trigger could for instance be an emotional state or specific situations. Self-reflection is prompted by examples of triggers and option to input experienced trigger. Users are subsequently prompted to identify alternative behaviour that could replace risk behaviours or habits. In addition, calculator tools are included which aim to raise awareness, for instance addressing distance and duration of active transport between home and school.

In addition to the content described above, users can opt for additional support via automated text messages. For alcohol, diet, and physical activity there are three text messages per week available, and users can at any time decide to subscribe or unsubscribe to these messages. The text messages intend to inform, support, and encourage users to maintain health behaviour change, and are based on our previous research [13, 16, 33–37]. Finally, a more comprehensive text message program is available to support smoking cessation for those users who wish to have extra support, which has been evaluated previously in a randomised controlled trial [38]. A key principle of health promotion according to Bandura is to equip youths with skills and efficacy beliefs that increases their ability to manage emotional and social pressures [39]. Thus, the components of LIFE4TOYth consist of different approaches, each aimed at increasing end-users’ belief in their ability to adopt new, healthy behaviours. Please see Additional file 2 for further details.

#### Control

Participants randomised to the control condition will be told that they will go through an initial phase of 4 months during which they are to increase their motivation and change their lifestyle on their own, after which they will receive additional support in form of the novel intervention. Furthermore, control condition participants will be recommended to visit a national website with general lifestyle and health information (https://www.1177.se/liv--halsa/).

#### Randomisation

Block randomisation using random selection of block sizes of 2 and 4 will be used to ensure 1:1 randomisation without creating risk for revealing the allocation sequence. All randomisation sequences will be computer generated and allocation will be automatically done by the backend system. While research personnel will be blind to participant allocation, participants will be aware of which condition they receive, thus the trial will be single blinded.

### Outcomes

#### Measures

Outcomes are listed here and subsequently explained. All questionnaires used in the trial can be found in Additional file 1.

**Primary outcome measures**

- **Alcohol:** Weekly alcohol consumption; monthly frequency of heavy episodic drinking.
- **Diet:** Average daily consumption of fruit and vegetables; weekly consumption of sugary drinks.
- **Physical activity:** Weekly moderate to vigorous physical activity (MVPA).
- **Smoking:** Four week point prevalence of smoking abstinence.

**Secondary outcome measures**

- Weekly consumption of candy and snacks.
- Body mass index (BMI).
- Number of cigarettes smoked weekly.

**Mediation measures**

- Confidence in one’s ability to change; importance of change; knowledge of how to change.

#### Primary and secondary outcome measures

Weekly alcohol consumption will be assessed by asking participants about the number of standard drinks of alcohol they consumed in the last week (short term recall method [40]). Using a short time span allows us to use a summary measure rather than day-by-day without any noteworthy bias [41]. Frequency of heavy episodic drinking will be assessed by asking participants how many times they have consumed more than four standard drinks of alcohol on one occasion in the past month. These two outcomes are both part of the proposed core outcome set for brief alcohol interventions [42, 43].

Dietary and physical activity variables are assessed by means of a modified version of questionnaires published by the National Board of Health and Welfare in Sweden [44]. Weekly consumption of fruit and vegetables will be assessed by asking two questions regarding how many portions (100 g) of fruit and vegetables (respectively) participants consumed on average per day during the past week. Consumption of sugary drinks will be assessed by asking participants how many units (33 cl corresponding to 1 standard can) of sugary drinks they consumed the past week. MVPA will be assessed by summing responses to two questions regarding the number of minutes spent on physical activity in the past week (moderate and vigorous respectively).

Body mass index will be measured by asking participants to report their weight at follow-up (height has been reported at baseline and is unlikely to have changed significantly).

Four week point prevalence of smoking abstinence (no cigarettes the past week) will be asked as a binary question. This is a suggested measure by the Society of Research on Nicotine and Tobacco [45]. Participants who have smoked any cigarette the past 4 weeks will be asked for the number of cigarettes smoked the past week.

#### Mediation measures

To further understand how the intervention may affect behaviour change, self-efficacy, perceived importance, and know-how (or possessed skills) of and for behaviour change [46–50] will be measured. These measures will be used to estimate to which degree the total effect of the intervention is mediated through these factors. Confidence, importance, and know-how will be measured by asking “How confident are you that you will be able to change your lifestyle?”, “How important do you think it is to change your lifestyle?” and “How well do you know how to change your lifestyle?” all three will have response options on a 10-point scale. We decided not to use validated measure of these three constructs to reduce participant burden, as attrition is problematic in general for digital intervention studies and in particular for this population.

#### Exploratory measures

Apart from the primary, secondary and mediation measures, the trial will also explore the acceptability of the intervention, and the reactions and actions of participants allocated to the control group. Acceptability will be measured using a five-item questionnaire regarding the experience of receiving support (intervention group only, please see Additional file 1). These five items measure participants’ view on perceived support received, specific components of the novel intervention, and if they would recommend the intervention to others. These we found could not be captured with validated measure of acceptability or usability such as the intervention appropriate measure [51] or system usability scale [52]. The two-item questionnaire capturing the control group’s reactions and actions was chosen to reduce participant burden and based on questions we have used in previous trials [13, 33, 53].

#### Follow-up

Follow-ups will be initiated by sending text messages to participants with hyperlinks to questionnaires at 2 and 4 months after randomisation. In all cases, the following attempts will be made to collect data:

1. A total of two reminders will be sent 2 days apart to those who have not responded.
2. If there is no response given, we will attempt to call participants to collect responses for the primary outcome measures only. A maximum of 5 attempts will be made

### Statistical analysis

Analyses will be done keeping participants within the groups to which they were randomised. Analyses will be conducted using both available data and missing data imputed (multiple imputation with chained equations). We will conduct attrition analyses to explore the missing at random (MAR) assumption underlying these analyses. First, if data is missing systematically then it may be the case that early responders differ from non-responders, and in extension that late responders are more like non-responders. Therefore, one analysis will regress primary outcomes against number of attempts to collect follow-up before a response was recorded. Second, we will investigating if responders and non-responders are different with respect to baseline characteristics.

Data will be graphically examined for outliers or data input errors, and sensitivity analyses will be performed excluding any erroneous data points.

Longitudinal data will be analysed using multilevel models with adaptive intercepts (per individual) and time by group interactions. Bayesian inference will be used to estimate the parameters of the models [54–57] with standard normal priors. For each group by time coefficient, we will report the marginal posterior probability of effect, and the median will be used as a point estimate of the magnitude of the effect. We will also report 50 and 95% compatibility intervals. We will complement the Bayesian analyses with maximum likelihood estimates and null hypothesis test at the 0.05 significance level. Both Bayesian and maximum likelihood estimates will be used for scientific inference.

### Models

#### Primary and secondary outcomes

Analyses of primary outcomes will be conducted among those fulfilling the respective criteria for inclusion at baseline, for example weekly alcohol consumption will be analysed among those who reported having consumed 10 or more drinks of alcohol in the past week. BMI and candy/snacks will be analysed among all participants, and number of cigarettes smoked weekly among baseline smokers.

Weekly alcohol consumption, frequency of heavy episodic drinking per month, weekly intake of candy and snacks, number of sugary drinks per week, and cigarettes smoked per week are all count variables that are likely skewed and overdispersed. Therefore, these outcomes will be analysed using negative binomial regression. If found not to be overdispersed, we will consider using normal regression (possibly log transformed). Average intake of fruit and vegetables per day, MVPA minutes per week, and BMI will be analysed using normal regression (possibly log transformed). Point prevalence of smoking abstinence will be analysed using logistic regression.

All models will be adjusted for sex, age, family’s economic situation, and mediator variables at baseline. Effect modification will be investigated by estimating the outcome models with interaction terms for each baseline variable respectively. To further investigate effect modification with respect to socioeconomic status, we will estimate the outcome models with both education and economic status as interaction terms. A final effect modification model will be estimated with a binary interaction variable representing age being greater to or equal to 18 (ie. drinking age).

#### Mediator outcomes

Mediators will be explored using a causal inference framework [58, 59], where Monte Carlo methods are relied upon for inference. This allows for any type of model (linear and nonlinear) to be used to represent the relationships between the group allocation, mediating variable, and the outcome. Four models will be created for each primary outcome, three which investigate the mediating factors on their own, and a fourth which incorporates all mediators at once. If any baseline characteristics are found to moderate the effect in the primary analysis, then additional mediator models will be created to include these as moderators.

### Exploratory outcomes

#### Interactions among lifestyle change

Outcome interactions, and determinants of such, will be investigated in an exploratory analysis. For instance, those who quit smoking may also be more likely to reduce their alcohol consumption, and this interaction may be moderated by baseline characteristics. Models to detect such interactions will be explored and findings will be used to create hypotheses for future research.

#### Protective effects of the intervention

The primary analyses are all done among those presenting with particular health risk behaviours at baseline, however, all participants will receive the same multiple lifestyle intervention, thus it is possible that the intervention may protect participants from developing unhealthy behaviour. To investigate this we will, for each primary outcome, contrast all participants in the intervention and control groups to assess if either group is more or less likely to develop unhealthy risk behaviours over the intervention period.

#### Dosage-response

Data collected week-by-week in the intervention group may be useful to identify trends in potential behaviour change over time. Exploratory models will be created to identify patterns that are informative about intervention effects (such as plateaus). Similarly, we will regress primary outcomes on usage statistics in the intervention group, including frequency of use of different modules and whether or not the participant decided to stop the intervention before the end of the trial, possibly identifying a dose-effect relationship.

#### Heterogeneous treatment effects

Randomised controlled trials traditionally contrast two or more groups, however do not address individual variability (known as heterogenous treatment effects [60]). Some individuals may respond well to an intervention, while others might not, and some may be harmed - however contrasting two groups does not identify such individual level differences. To expand upon the effect modification analyses done in the primary analyses, we will explore prediction models which we will use to predict the outcome for each trial participant given both randomised conditions. Calibration will be assessed using cross-validation. The model will be used to predict how much each individual would benefit (or be harmed) by the novel intervention, and clustering will be used to identify groups of participants that are affected equally by the intervention. We will then explore similarities within each cluster of participants (for instance through multinomial regression) to identify which baseline characteristics are associated with benefit and harm of the intervention.

#### Power calculations

Considering the novelty of the intervention (digital multiple lifestyle) and the population (Swedish high school students), there are no existing full-scale trials from which an estimate of effects can be assumed. Also, as there are no interventions of this type available to high school students in Sweden, minimal relevant effect sizes were considered for this trial. We have based expected effect sizes on what we believe are minimally relevant for the population, and reasonable, given the target population and digital intervention context. For those included based on weekly alcohol consumption, who drink 10 or more standard drinks per week, we believe a minimal relevant effect size to be a difference in means at follow-up of 3 standard drinks per week. Similarly, those who are included based on having one episode of heavy episodic drinking per month, a minimal relevant difference at follow-up would be 0.3 less episodes on average per month (or about 1 per 3 months). For physical activity, participants are included if they have less than approximately 60 min of MVPA per day, for which a mean difference at follow-up of 30 min per week (i.e., 5 min per day) would be a minimal effect size of importance in this group. Following the same reasoning as above, among those included based on eating less than 500 g of fruit and vegetables on average per day, we believe a minimal relevant effect size to be a difference in means of 0.5 portions per day (50 g per day). Among those included based on sugary drinks consumption (3 or more drinks per week), we believe that a mean difference of 2 drinks per week at follow-up represents a minimal effect size. Finally, for smoking, we rely on data from a text message smoking cessation which was evaluated among Swedish high school students, suggesting that we may expect approximately 10% less smokers in the intervention group.

Using the minimally relevant effect sizes described above, we conducted a Monte Carlo study to guide recruitment. The study was designed to identify the necessary number to recruit for each of the six outcomes in order to achieve a power of 80% at the 0.05 significance level. Recruitment will continue until each of the six outcomes have been powered (no more than 24 months). All assumed effects were allowed to vary during the Monte Carlo simulations, following a normal distribution with a standard deviation of 0.025.

Based on attrition observed in a previous trial of a digital smoking cessation intervention in this population [61], we assumed 25% attrition to follow-up. We found that we will require 267 individuals for weekly alcohol consumption, 534 for heavy episodic drinking, 734 for sugary drinks, 334 for fruit and vegetables, 800 for MVPA, and 467 for smoking. Thus, we will recruit until all of these number have been achieved (or 24 months have passed). Using data from surveys on Swedish high school students’ health behaviour, we found that this will likely require the recruitment of 2000 students to the trial, although correlations among behaviours may reduce this overall number.

## Discussion

This study considers the “big four” health behaviours, i.e. alcohol, diet, physical activity, which all have a strong impact on health. As a growing body of research suggests that these health risk behaviours typically cluster and do not occur in isolation [21–23, 62], this trial can offer needed knowledge on the feasibility and effectiveness of an mHealth intervention targeting multiple behaviours, and to provide an mHealth platform where users can navigate freely using a personal dashboard that provides access to the four behaviour modules. The combination of recurring screening, text messages, and an interactive platform also mean a flexibility that encourage individual preferences. Findings in a systematic review and meta-analysis that evaluated the effectiveness of mHealth interventions targeting adolescents suggested that interventions using multiple mHealth solutions such as a combination of mobile applications, text messages and phone calls, have better potential than interventions that use single mode of delivery such as phone calls for instance. However, the results in the review was inconsistent between different outcomes which mean an uncertainty regarding the effectiveness of different mHealth solutions [63]. Therefore, the current study might contribute to the understanding of the effectiveness of interventions that use a combination of a mHealth solutions such as automatic text messages and interactive dashboard platforms.

### Limitations

While almost all of the trial processes are automated, one potential risk of detection bias is the use of follow-up by telephone among those not responding to initial automated attempts. While every effort will be taken to avoid prompting participants to reveal such information, participants may disclose their group allocation to research personnel at this stage. Overall, we believe that the benefits of decreasing follow-up attrition by calling non-responders reduces the risk of bias from missing data and outweighs this risk of detection bias.

We are expecting to have relatively low attrition, due to a scheme of collecting follow-up data which has been successful in our previous studies [13, 33–35], despite not incentivizing participants. However, our power calculation is based on a Monte Carlo study, which takes into consideration uncertainty in our estimates, thus we expect that our calculations will be robust to slight deviations from assumptions.

The use of non-validated questionnaires for measuring mediators, acceptability, and experience of the intervention and control groups is also a limitation of this study. The decision to do so is based on reducing participant burden (to avoid attrition), but also to capture dimensions which are not present in validated measures. This does however limit both comparison with other studies, and the degree to which we can credit mediated effects to specific psychosocial constructs, as the face-valid single items are not validated to do so.

Finally, by randomising participants on individual level, rather than school level, we could potentially increase the risk of contamination between treatment groups. The risk of contamination is commonly present in digital intervention trials, as information is easily shared among participants. Cluster randomisation may reduce the risk; however, it may also create false confidence that the risk has been mitigated. High school students in Sweden are divided into something that resembles a traditional school class; however, students are mixed across classes and schools when attending courses. Young adults’ presence on social platforms also removes any geographical limitation that could be used for clustering; thus, there is no randomisation level that would sufficiently shield participants. In addition, distance learning has been implemented at times throughout the Covid-19 pandemic, which also limits interactions. Clustering would, therefore, in this case only accomplish a false sense of bias reduction. Analysis by treatment allocation, disregarding potential contamination, will bias estimates towards the null, potentially resulting in more conservative estimates than can be expected in a full-scale roll-out.

## Summary

Only one in three Swedish high school students fulfil physical activity recommendations, and most eat too little vegetables and fruit and too much sweets and savoury snacks [64]. Heavy episodic drinking and smoking continues to be a problem among high school students, despite a nationwide decline in smoking prevalence [7, 8]. The findings of this trial could be generalised to a diverse high-school student population as our recruitment encompass a large proportion of schools throughout Sweden with various educational profiles. Furthermore, if effective, the mHealth intervention has good potential to be able to be scaled up and disseminated at high schools nationally. The intervention could potentially aid those who seek to change their health behaviour on their own and could reduce the burden of disease from noncommunicable diseases in Sweden.

## Supplementary Information

**Additional file 1.** Questionnaires.

**Additional file 2.**

## Data Availability

Not applicable.

## Abbreviations

MVPA: Moderate to vigorous physical activity
BMI: Body mass index
MCAR: Missing completely at random
