# Supplementary material for: mHealth intervention for multiple lifestyle behaviour change among high school students in Sweden (LIFE4YOUth): protocol for a randomised controlled trial
Source: BMC Public Health. 2021 Jul 16;21:1406. doi: 10.1186/s12889-021-11446-9 (PMC8283383; doi:10.1186/s12889-021-11446-9)
Supplement: Supplementary file 2 — Additional file 2. [file 12889_2021_11446_MOESM2_ESM.docx]

| Theoretical basis for the LIFE4YOUth intervention including theoretical construct, examples of intervention content and corresponding behaviour change technique [1]. | | |
| --- | --- | --- |
| **Construct** | **Behaviour change technique** | **Example of intervention content** |
| Knowledge | Feedback on behaviour  Self-monitoring of behaviour  Information about health consequences  Information about social and environmental consequences | Tailored feedback on health risks.  Factual information about health consequences of risk behaviours. |
| Self-efficacy | Instruction on how to perform a healthy behaviour | Accessible tips and strategies to try out. |
| Outcome expectations | Incompatible beliefs  Salience of consequences | Prompts to explore reasons for behaviour change through a series of predefined statements. In addition, a calculator tool to highlight consequences (e.g., financial) of risk behavior. |
| Goals | Goal setting (behaviour) | A checklist including explanations and examples to promote well-defined goals. |
| Perceived facilitators | Problem solving  Action planning | Prompts and exercises to reflect on current everyday habits to increase the understanding of the formation of habits. |

[1] Michie S, Richardson M, Johnston M, Abraham C, Francis J, Hardeman W, Eccles MP, Cane J, Wood CE. The behavior change technique taxonomy (v1) of 93 hierarchically clustered techniques: building an international consensus for the reporting of behavior change interventions. Ann Behav Med. 2013;46(1):81-95.
